# Deep learning algorithms for detecting and visualising intussusception on plain abdominal radiography in children: a retrospective multicenter study

**DOI:** 10.1038/s41598-020-74653-1

**Published:** 2020-10-16

**Authors:** Gitaek Kwon, Jongbin Ryu, Jaehoon Oh, Jongwoo Lim, Bo-kyeong Kang, Chiwon Ahn, Junwon Bae, Dong Keon Lee

**Affiliations:** 1 Department of Computer Science, Hanyang University, Seoul, Republic of Korea; 2 Department of Software and Computer Engineering, Ajou University, Suwon, Gyeonggi Do Republic of Korea; 3 Department of Emergency Medicine, College of Medicine, Hanyang University, Seoul, Republic of Korea; 4 Machine Learning Research Center for Medical Data, Hanyang University, Seoul, Republic of Korea; 5 Department of Radiology, College of Medicine, Hanyang University, Seoul, Republic of Korea; 6 Department of Emergency Medicine, College of Medicine, Chung-Ang University Hospital, Seoul, Korea; 7 Department of Emergency Medicine, Seoul National University Bundang Hospital, Gyeonggi-do, Republic of Korea

**Keywords:** Gastroenterology, Medical research, Mathematics and computing

## Abstract

This study aimed to verify a deep convolutional neural network (CNN) algorithm to detect intussusception in children using a human-annotated data set of plain abdominal X-rays from affected children. From January 2005 to August 2019, 1449 images were collected from plain abdominal X-rays of patients ≤ 6 years old who were diagnosed with intussusception while 9935 images were collected from patients without intussusception from three tertiary academic hospitals (A, B, and C data sets). Single Shot MultiBox Detector and ResNet were used for abdominal detection and intussusception classification, respectively. The diagnostic performance of the algorithm was analysed using internal and external validation tests. The internal test values after training with two hospital data sets were 0.946 to 0.971 for the area under the receiver operating characteristic curve (AUC), 0.927 to 0.952 for the highest accuracy, and 0.764 to 0.848 for the highest Youden index. The values from external test using the remaining data set were all lower (P-value < 0.001). The mean values of the internal test with all data sets were 0.935 and 0.743 for the AUC and Youden Index, respectively. Detection of intussusception by deep CNN and plain abdominal X-rays could aid in screening for intussusception in children.

## Introduction

Intussusception is an acquired invagination of the proximal segment of the intestine into the distal segment and is the most common cause of intestinal obstruction among children aged 3 to 36 months old^1–3^. This disease is a relatively common cause of emergency room visits in children. Rapid diagnosis and treatment with air enema within 24 h from the onset can alleviate symptoms in approximately 84% of patients; however, prolonged cases can develop ischaemia, necrosis, and perforation^4,5^.

There are several imaging studies available for diagnosing intussusception. Hydrostatic or pneumatic enemas were considered the gold standards for both diagnosing and treating intussusception^6^. However, these are invasive radiologic procedures that must be performed by radiologists and are not always readily available^7^. Conversely, ultrasonography has been proven to be a reliable first-line diagnostic modality for patients suspected to have intussusception^8–10^. However, the utility of this procedure is affected by the skill of the operator and variations in equipment—the availability of which may be limited in certain areas. Plain abdominal radiography is inexpensive and is commonly used as a first-line screening test for intussusception in patients with gastrointestinal signs and symptoms^11,12^. Despite its low sensitivity (< 50%) and poor rate of inter-observer agreement in diagnosing intussusception, it remains an important diagnostic modality and has long been used to screen for other diseases such as constipation, ileus, and peritoneal air^6,12,13^.

Deep convolutional neural networks (CNN) are used for widespread image detection and classification and have been utilised in the fields of radiology and medical image analysis^14–17^. An automated method for screening plain abdominal radiographs and prioritising positive images for rapid review and diagnosis may minimise possible delays in diagnosing intussusception and reduce the incidence of misdiagnoses; this is especially important in medical environments, such as primary care institutions, where there is little or no knowledge of intussusception during emergency situations. Deep CNN models (1) require large and well-curated training data sets that contain significant visual heterogeneity, (2) must be tested through external validation, and (3) must undergo optimisation of equipment and settings to ensure high accuracy and performance in various clinical environments^17^. There are no previous studies on the availability and external validity of deep learning in diagnosing intussusception using large data sets of plain abdominal radiographs. This study aimed to create a human-annotated data set of plain abdominal X-rays of children with intussusception for internal and external validation, and to verify a possibility of deep CNN to detect intussusception with this set.

## Results

**Figure 1:**
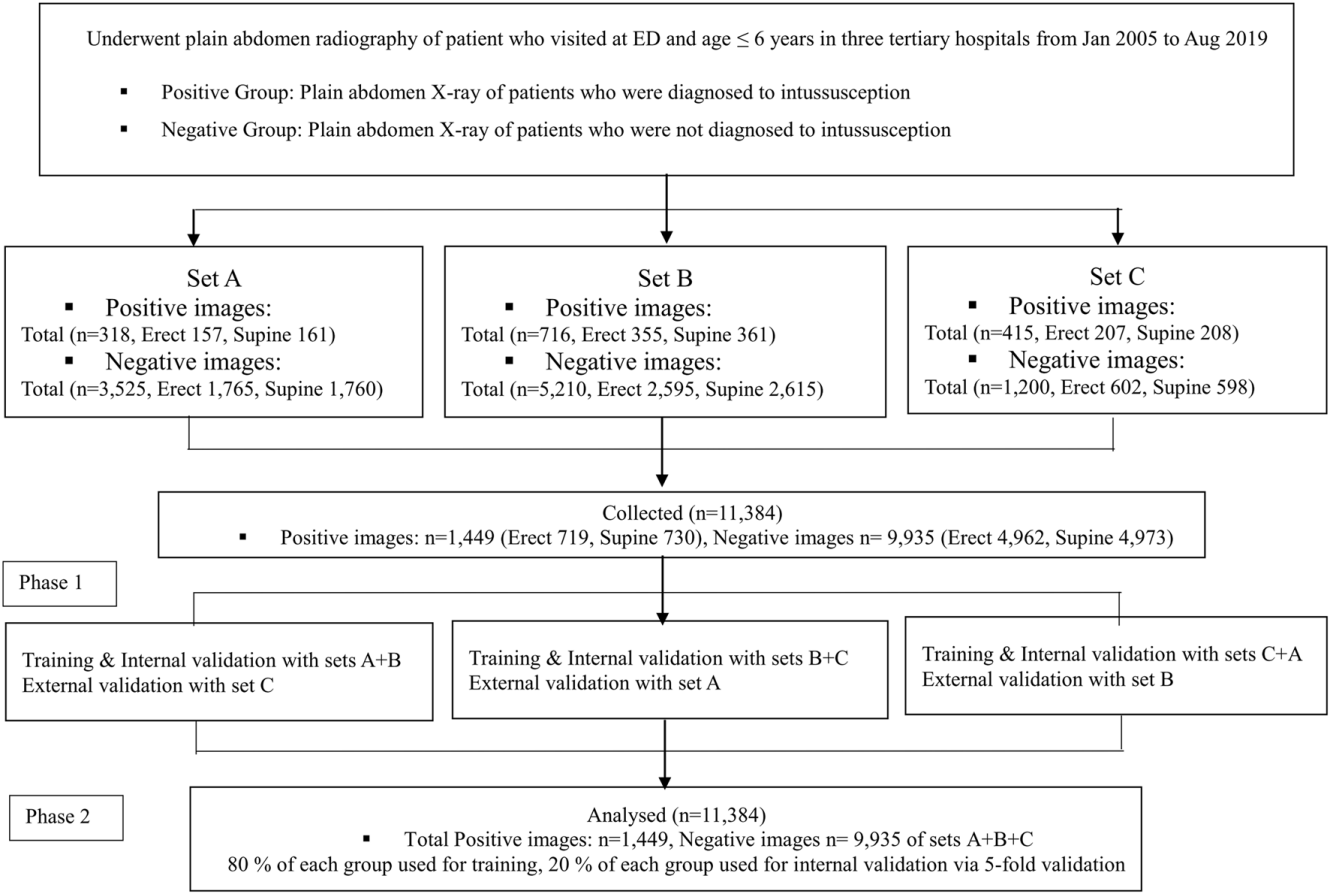
Flow chart of data collection and analysis. ED, emergency department.

A total of 11,384 images consisting of 1449 positive images and 9935 negative images were collected (Fig. 1). The baseline characteristics of participants who provided these images are shown in Table 1. Significant differences between the two groups (positive and negative image groups) were observed regarding age and sex in the sets gathered from hospitals B and C but not from the set provided by hospital A.

**Table 1:** Baseline characteristics of participants who provided images for the data sets.

	Positive images	Negative images	P-value
**Set A (n = 3843)**			
Images, n	318	3525	
Participants, n	161	1760	
Age, months, mean [s.d.]	21.8 [12.0]	24.0 [17.6]	0.12
Sex, male, n (%)	101 (62.7)	1207 (68.6)	0.13
**Set B (n = 5926)**			
Images, n	716	5210	
Participants, n	361	2615	
Age, months, mean [s.d.]	22.2 [17.9]	32.8 [17.9]	< 0.001*
Sex, male	228 (63.2)	1461 (55.9)	0.01*
**Set C (n = 1615)**			
Images, n	415	1200	
Participants, n	208	602	
Age, months, mean [s.d.]	20.9 [16.8]	31.1 [23.8]	< 0.001*
Sex, male	136 (65.4)	305 (50.7)	< 0.001*
**All (n = 11,384)**			
Images, n	1449	9935	
Participants, n	730	4977	
Age, months, mean [s.d.]	21.7 [15.8]	29.5 [19.3]	< 0.001*
Sex, male	465 (63.7)	2973 (59.7)	0.04*

Continuous variables are presented by mean [standard deviation] and categorical variables are presented by N (%), p < 0.05.

The independent t-test or the Kruskal–Wallis test were used to compare positive and negative groups according to normality. Categorical variables were presented as numbers and percentages and analysed using a chi-squared test.

*P-values < 0.05 were considered statistically significant.

### Phase 1: Training evaluation and internal validation tests using two data sets and external validation tests using the excluded data set

**Figure 2:**
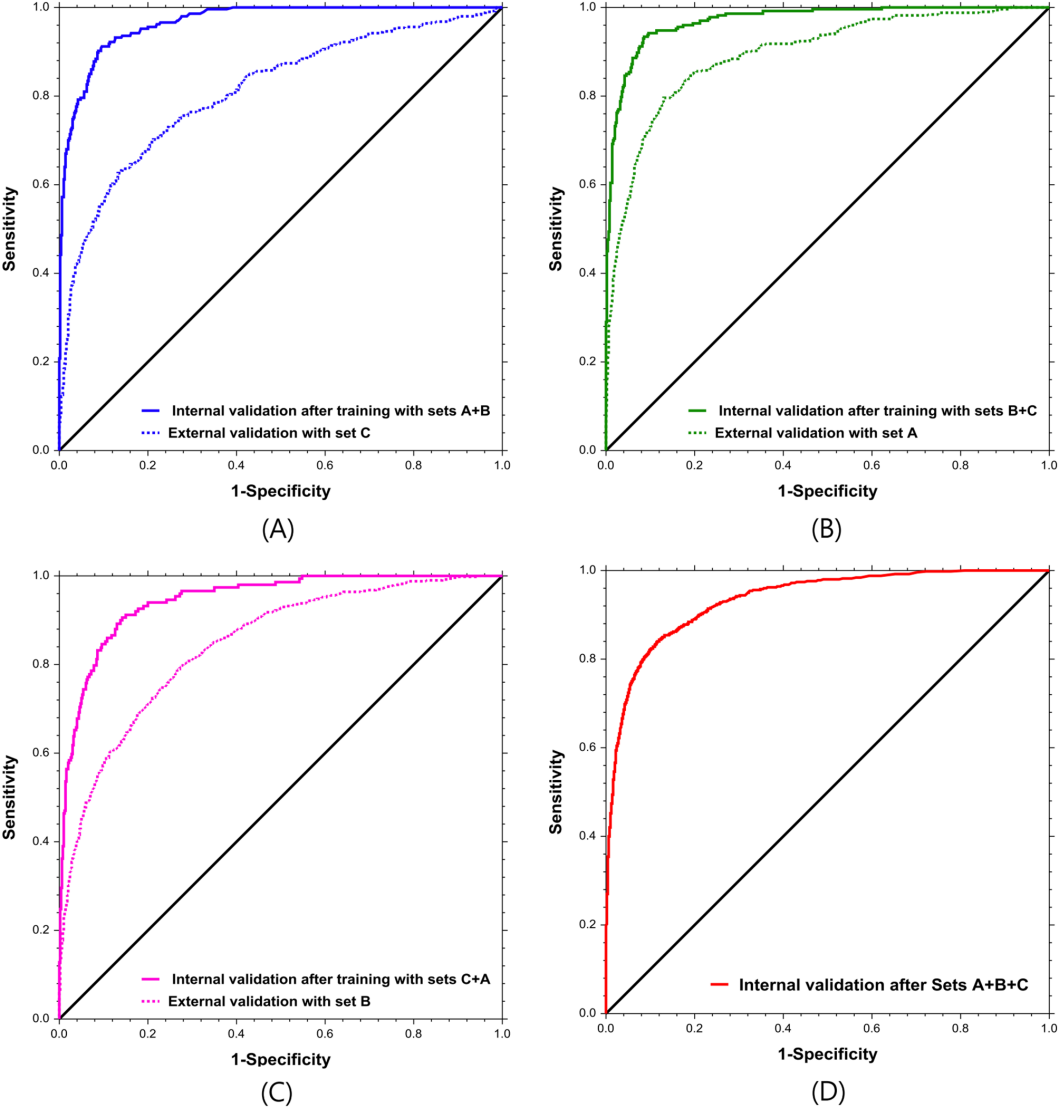
Receiver operating characteristic (ROC) curves of internal and external validation tests in Phase 1 and 2 experiments. (**A**) External validation with set C after training and internal validation with sets A + B, (**B**) External validation with set A after training and internal validation with sets B + C, (**C**) External validation with set B after training and internal validation with sets C + A, (**D**) Internal validation after training with sets A + B + C.

The diagnostic performance matrix of the internal and external validation tests, including the optimal cut-off values, are shown in Table 2. The values of the internal validation test after training with data sets A + B, B + C, and C + A were: 0.966 (0.955, 0.975), 0.971 (0.959, 0.980), and 0.946 (0.926, 0.961), respectively, for the AUC (95% CI); 0.952, 0.943, and 0.927, respectively, for the highest accuracy; and 0.818, 0.848, and 0.764, respectively, for the highest Youden index. The values of the external validation test using the excluded sets (external validation of set C as the counterpart of the internal validation test using sets A + B, etc.) were: 0.811 (0.784, 0.835), 0.895 (0.874, 0.913), and 0.844 (0.828, 0.858), respectively, for AUC; and 0.421, 0.431, and 0.493, respectively, for the Youden indices. All values had a P-value < 0.001 (Table 3, Fig. 2).

**Table 2:** Diagnostic performance matrix of the internal and external validation tests with optimal cut-off values (Phase 1).

	(A)	Positive	Negative	(B)	Positive	Negative	(C)	Positive	Negative
Internal validation	Predicted positive	188	166	Predicted positive	214	122	Predicted positive	136	141
	Predicted negative	18	1581	Predicted negative	13	1161	Predicted negative	13	805
External validation	Predicted positive	329	446	Predicted positive	301	1817	Predicted positive	466	822
	Predicted negative	86	754	Predicted negative	17	1708	Predicted negative	250	4388

The optimal cut-off value was estimated based on the highest Youden index in the internal validation tests.

(A) External validation test with set C set after training and internal validation test with sets A + B, (B) External validation with set A after training and internal validation with sets B + C, (C) External validation with set B after training and internal validation with sets C + A set. Positive; intussusception, Negative; no intussusception. Youden Index is the Sensitivity + Specificity − 1.

**Table 3:** Outcomes of the internal validation test after the training with two data sets and of the external validation test using the excluded data set (Phase 1).

	Training and internal validation test							External validation test					P-value of difference between two validation (95% CI)
	Data set	AUC	Highest accuracy	Highest Youden index	Sen	Spe	Optimal cut-off value	Data set	AUC	Youden index	Sen	Spe	
(A)	A + B	0.966 (0.955, 0.975)	0.952	0.818	0.913	0.905	0.02	C	0.811 (0.784, 0.835)	0.421	0.793	0.628	< 0.001* (0.128, 0.183)
(B)	B + C	0.971 (0.959, 0.980)	0.943	0.848	0.943	0.905	0.06	A	0.895 (0.874, 0.913)	0.431	0.947	0.485	< 0.001* (0.059, 0.102)
(C)	C + A	0.946 (0.926, 0.961)	0.927	0.764	0.913	0.851	0.01	B	0.844 (0.828, 0.858)	0.493	0.651	0.842	< 0.001* (0.080, 0.125)

(A) External validation with set C after training and internal validation with sets A + B, (B) External validation with set A after training and internal validation with sets B + C, (C) External validation with set B after training and internal validation with sets C + A, (D) Internal validation after training with sets A + B + C. Positive, with intussusception; negative, without intussusception. AUC, area under the receiver operating characteristic curve (ROC). Accuracy, the fraction of the correct predictions over the total number of predictions. The Youden index, sensitivity + specificity – 1—that is, the vertical distance between the 45° line and the point on the ROC curve. In the external validation tests, we selected the optimal cut-off value based on the highest Youden index value in the internal validation tests. CI, confidence interval. Sen, sensitivity. Spe, specificity.

*P-values < 0.05 indicate a statistically significant difference.

### Phase 2: Internal validation test using all data sets

The results of the internal validation tests are summarised in Table 4. The mean values (95% CI) gathered from the internal validation tests were 0.935 (0.928, 0.941) and 0.743 (0.722, 0.763) for the AUC and the highest Youden index, respectively. The mean values for sensitivity and specificity were 0.816 (0.789, 0.842) and 0.925 (0.893, 0.957), respectively, in the highest Youden index. (Table 4, Fig. 2).

**Table 4:** Outcomes on the internal validation test after training with all data sets (Phase 2).

Test	Outcomes			AUC (95% CI)
	Highest Youden index	Sen	Spe	
1st	0.737	0.816	0.921	0.936 (0.918–0.950)
2nd	0.731	0.784	0.936	0.946 (0.931–0.958
3rd	0.760	0.817	0.943	0.949 (0.934–0.961)
4th	0.726	0.844	0.882	0.922 (0.904–0.937)
5th	0.760	0.817	0.943	0.949 (0.934–0.960)
Mean (95% CI)	0.743 (0.722–0.763)	0.816 (0.789–0.842)	0.925 (0.893–0.957)	0.935 (0.928–0.941)

AUC, area under the receiver operating characteristic curve (ROC). The Youden index, the sensitivity + specificity – 1—that is, the vertical distance between the 45° line and the point on the ROC curve. In the internal validation tests, after training with all data sets, we selected the outcome values based on the highest Youden index.

CI, confidence interval. Sen, sensitivity. Spe, specificity.

**Figure 3:**
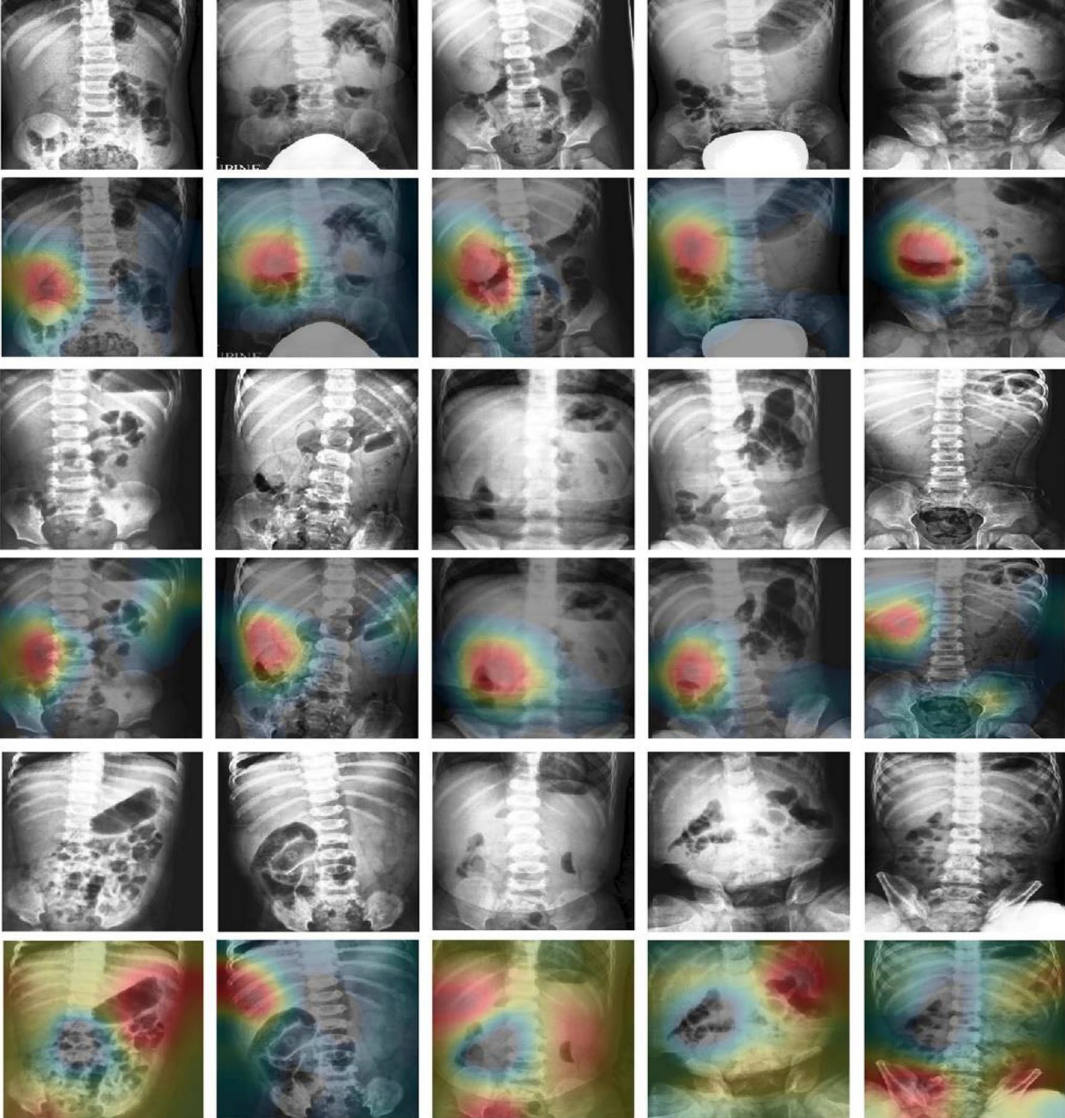
Class activation map (CAM) for images which were true positive in the 2nd internal validation test using all data sets. The images in the odd row are the original images while those in the even row are images with CAM applied. Unidentified areas was highlighted by CAM in images from the 6th row, whereas it highlighted the correct areas in the 2nd and 4th rows.

We visualised the feature maps of images from the second internal validation test where intussusception was detected with the highest Youden index (0.731). From the visualisation of 292 images, the correct area was chosen by the network in 255 cases, which indicates that the network has learned how to detect and classify intussusception. True positive images are shown in Fig. 3.

## Discussion

The classic triad of intussusception—red currant jelly stools, colicky abdominal pain, and vomiting—was seen in less than 40% of the children in this study; these nonspecific signs and symptoms make the diagnosis of intussusception challenging and force clinicians to rely only on the patient’s history and physical examination findings^18–20^. Point-of-care ultrasound, when performed by an emergency medicine physician, has a high diagnostic accuracy for intussusception, with sensitivity and specificity values of 0.94 and 0.98, respectively; these results are similar to those of radiologist-performed ultrasounds^21^. Ultrasound is easy for other physicians—even novice ones—to perform, and it allows minimisation of radiation exposure for the patient. However, because the mean annual intussusception incidence rate is approximately 30 per 100,000 live births in the first 3 year of life^3^, using ultrasound as a screening exam to rule out intussusception in all children who present with nonspecific signs and symptoms is difficult.

In a study on the use of risk stratification in evaluating intussusception in children, it was found that abdominal radiography could be used as the initial diagnostic modality to identify children at risk with sensitivity and specificity values of 0.77 and 0.79, respectively^22^. However, these radiographs were interpreted by paediatric radiologists using predefined criteria such as small bowel obstruction, target or crescent signs, and findings consistent with ileocolic intussusception. Kim et al. reported that drawing rectangular ROI indicators on abdominal radiographs could allow deep learning-based algorithms to aid in screening for right upper quadrant ileocolic intussusception in young patients. According to a 75-image internal validation test, the sensitivity and specificity values of their algorithm were 0.76 and 0.96, respectively, which are better than those of a radiologist who was found to have sensitivity and specificity values of 0.56 and 0.92, respectively^23^. In our study, we drew a rectangular ROI that encompassed the entire abdomen; the ranges of the sensitivity and specificity values after conducting training and internal tests using two data sets were 0.913–0.943 and 0.851–0.905, respectively. In a study on the use of deep learning for diagnosing small bowel obstruction using plain abdominal radiography, the detection accuracy was found to significantly improve with the number of positive training radiographs used^24^. We believe that our algorithm, which used a large volume of data, improved the outcomes of using deep learning to detect intussusception. The application of this deep learning-based algorithm as a screening tool in the hospitals that provided the data sets used can decrease the unnecessary use of abdominal ultrasonography.

The AUC and Youden index values from all three external validations that were performed were found to be lower by approximately 0.15 and 0.4, respectively, than the values from the internal test. Possible explanations for these findings include differences in data volume, variations in the proportion of positive and negative images, and differences in the quality of each data set. However, the sensitivity of the external validation test was higher by at least 0.65; this indicates that the completed model, which was trained using two hospital data sets, can be transferred to other hospitals and used as a screening tool for diagnosing intussusception. In internal validation tests with fivefold cross-checking and training with all sets, all values of the Youden index, including sensitivity and specificity, were higher than values from the external validation tests with other set after the training and internal validation with two sets. To optimise performance in specific environments, hospitals that will use the model must train it using their own positive and negative images. In our study used CAM for visualisation, we showed which part of the plain abdominal X-rays the model focused on.

There are several limitations to this study. First, we did not compare the performance of our model against that of physicians with respect to key factors such as clinical outcomes, the time required to arrive at a diagnosis, and the equipment needed to use the model as a screening tool. Second, we did not annotate the actual location of intussusception on the X-ray images. Thus, we trained deep CNN under weak supervision using only the existence of intussusception. Better performance can be expected with full supervision and coordinated information regarding the location of the intussusception. Third, there was a difference in resolution between the medical images and the input images of the deep CNN. The resolution of extracted medical images in our data set was approximately 3000 × 4000, while the resolution of input images for our model was only 224 × 224. Therefore, it is possible for information loss to occur when attempting to detect intussusception since the medical images were downsampled. However, if the image size is too large, both the number of computations and the size of the memory consumed increase exponentially; this might render the operation too slow or even impossible to perform. Therefore, further studies that minimise information loss by appropriate resizing of images or selection of only the ROI are needed. Fourth, differences of age and sex between negative and positive group could make other information including body shape and bone growth in images and influence the training and detection of intussusception with deep CNN. Fifth, we stored the images with 8-bit JPEG gray scale format. This process could cause degradation of the data, since the image intensity levels and contrast for details are reduced and removed. Finally, although the ratios of training datasets were equally assigned for positive and negative cases by mini-batch training, the imbalanced testing dataset would decrease reliability of testing results.

In conclusion, we verified a possibility of a deep CNN algorithm that consists of abdominal detection and intussusception classification networks using plain abdominal X-rays to help physicians screen for intussusception. This algorithm can be trained by hospitals that can provide images before being transferred to other hospitals and used to screen for intussusception in children.

## Methods

### Study design

We conducted a retrospective study at three tertiary academic hospitals (Seoul and Gyeonggi-Do, Republic of Korea) between October 2019 and January 2020 to evaluate the role of deep learning in diagnosing intussusception using plain abdominal X-rays. This study was approved by the Institutional Review Board (IRB) of Hanyang University Hospital (ref. no. HYUH 2019-06-015), the IRB of Hanyang University Guri Hospital (ref. no. GURI 2020-01-006), and the IRB of Seoul National University Bundang Hospital (ref. no. B-1907-555-102) and the requirement for informed consent were waived by the IRBs of Hanyang University Hospital, Hanyang University Guri Hospital, and Seoul National University Bundang Hospital. All methods and procedures were carried out in accordance with the Declaration of Helsinki.

### Data set

#### Plain abdominal X-rays of patients diagnosed with intussusception (positive images)

We gathered data on patients who were diagnosed with intussusception and treated with hydrostatic or pneumatic enema at the emergency room from the medical records of Hanyang University Hospital (set A) and Hanyang University Guri Hospital (set B) from January 2005 to August 2019, and from Seoul National University Bundang Hospital (set C) from January 2010 to August 2019. The inclusion criterion was age ≤ 6 years. We obtained the supine and erect views of plain abdominal X-rays in all eligible patients; these images were validated, and a diagnosis of intussusception was made by radiologists before an abdominal ultrasound was performed.

#### Plain abdominal X-rays of patients not diagnosed with intussusception (negative images)

The candidate images for inclusion in the negative group were identified using X-rays of patients of the same age who visited the emergency room with complaints of abdominal pain, vomiting, or diarrhoea that was not indicative of intussusception. Their reports were stated by radiologists as ‘unremarkable study’, ‘non-specific finding’, ‘rule out paralytic ileus’, or ‘rule out gastroenteritis’. We collected these images from the same hospitals and within the same time period.

The collected images had a positive-to-negative ratio of approximately 1:3–1:12. All candidate images were extracted in the Digital Imaging and Communications in Medicine (DICOM) format used by the picture archiving and communication system (PACS, Centricity, GE Healthcare, Milwaukee, WI, USA), using a custom-built automated image retrieval system. We stored the images in an 8-bit JPEG grayscale format.

### Abdominal detection and Intussusception classification

**Figure 4:**
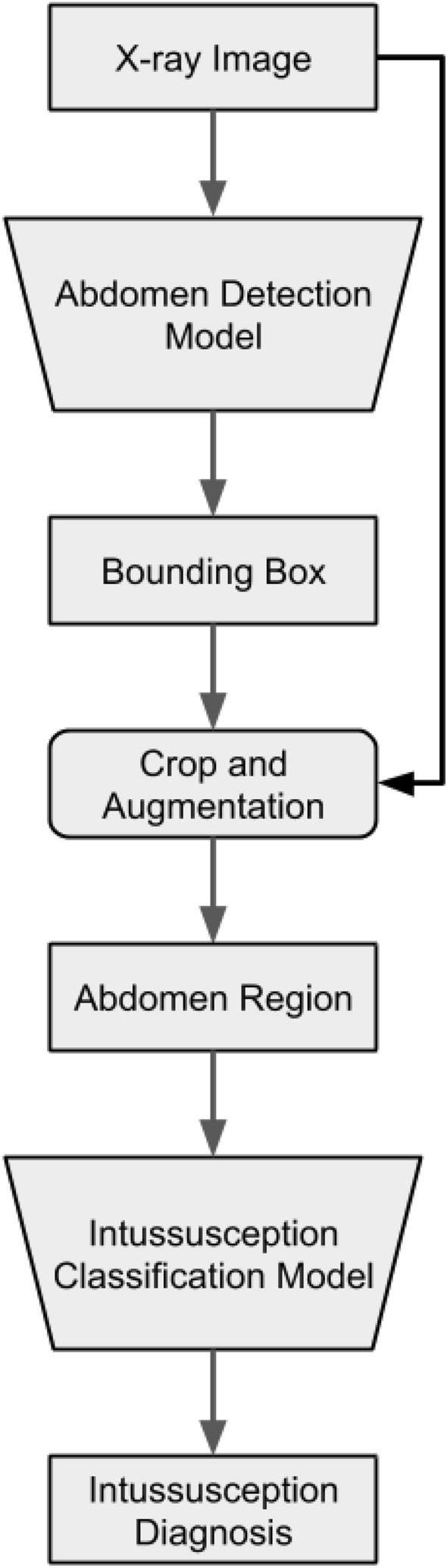
Intussusception screening system architecture. The proposed architecture consists of the abdomen detection model (top) and the intussusception classification model (bottom). The abdomen detection model detects the abdominal region from the entire X-ray image. The intussusception classification model detects intussusception on X-ray images that were cropped by the abdomen detection model.

The overall workflow of the proposed intussusception screening system is shown in Fig. 4. Our architecture consists of (1) an abdominal detection model that detects the abdominal region and (2) an intussusception classification model that detects intussusception.

#### Abdominal detection model

We used the Single Shot Multibox Detector (SSD) for the abdominal detection model^25^. The SSD generates default boxes with various ratios and scales from multiple feature maps to learn the regression model for object coordinates and the classification model for object label confidence. As we needed to detect the abdominal region, we changed the last fully connected layer to predict two classes: the abdomen and the background. Moreover, we retrained the last fully connected layer to compute the coordinates and confidence values for the abdominal region and the background. To train the abdominal detection model, we manually annotated the abdominal regions using Python 3.7 (https://www.python.org). Using the images of the patients’ abdomens, we selected rectangular regions of interest (ROI) spanning the diaphragm to the upper margin of the acetabulum along with the corresponding lateral borders.

#### Intussusception classification model

Among the deep learning CNN models for classification, which includes AlexNet, VGG, ResNet, and DenseNet, we used ResNet (Residual Network) as the intussusception classifier^26–29^. ResNet uses a skip connection that adds the input feature to the output of the residual layer. Because the skip connection allows the model to learn the difference between input and output features, it solves the gradient vanishing problem that occurs as the layer becomes deeper. Furthermore, we modified the last fully connected layer to predict the class probability of intussusception. A sigmoid activation function placed after the last fully connected layer normalised the class probability values to [0, 1]. The network weights were updated by the binary cross-entropy loss,

1 $$\text{BCE}\left(\text{x}\right)=-\sum_{i=1}^{C=2}\left[{y}_{i}logp\left(Y=i|X\right)+\left(1-{y}_{i}\right)\text{log}\left(1-p\left(Y=i|X\right)\right)\right],$$

where ${\text{y}}_{\text{i}}$ is the ground-truth label of the ${i}$th class in C ∈ {Intussusception, Normal}, and $\text{p}(\text{Y}=\text{i}|\text{X})$ denotes the probability for the ${i}$th class that the proposed method predicts for $\text{X}$ as the input X-ray image.

We used the MatConvNet deep learning library (version 1.0-beta25, https://www.vlfeat.org/matconvnet/) from MATLAB R2019b (https://mathworks.com/) to implement our detection and classification models. The trainings and tests were performed using a GTX Titan Xp GPU (NVIDIA, Santa Clara, CA, USA). The network weights were initialised from a pre-trained model on ImageNet^30^, and the network was trained end-to-end using stochastic gradient descent (SGD). We trained the model in batches of 16 with an initial learning rate of 0.001 that was linearly decreased over 100 epochs to 0.00001.

### Data augmentation and balanced training

Due to difficulties in acquiring large-scale medical images, effective augmentation of training data was needed to conduct robust training for the deep learning CNN. Although we collected approximately 11,384 images, which is not a small data size for evaluating the diagnostic capability of the algorithm, there remained immense potential to improve diagnostic performance through data augmentation. Therefore, we performed elaborate augmentations on the images by applying random rotation and translation changes. Overfitting problems would degrade diagnostic performance as the proportion of negative images was much higher than that of positive images. Thus, we sampled mini-batch training data that included the same number of positive and negative images to balance the training.

### Data experiments

We validated the performance of our method through two experimental phases. First, we used images from two of the three hospitals as the sets for training and internal validation tests, while the images from the other hospital were used as the external validation test set. The data from the two hospitals were separated as training (80%) and internal validation test (20%) data, to determine the optimal cut-off value for the external validation test. Since there were three hospital data sets, three cases of external validations were examined. Second, we performed training and internal tests using data from all three sets (A, B, and C). Eighty and 20% of each data set were used for training and internal validation tests via fivefold cross-validation, respectively. Any data used in these tests were excluded from the initial training data set.

The proposed method is a computer-aided diagnosis (CAD) system that assists radiologists and emergency physicians in analysing medical images. Therefore, it is better to show areas that are suspicious for intussusception rather than simply determining whether the input X-ray image is a case of intussusception or not. To intuitively identify intussusception, we visualised which areas of the X-ray image were predicted to contain the diagnosis using class activation maps (CAMs)^31^. To generate CAMs, we extracted the activation map, ${\text{f}}_{\text{k}}$, before the last global average pooling layer of the intussusception classification model. When the intussusception classification model determined the input X-ray image as intussusception, CAMs were obtained by multiplying the extracted activation map, ${\text{f}}_{\text{k}}$, with the weight in the final classification layer for the feature map k leading to pathology y ${\text{w}}_{\text{k}}$

2 $${\text{M}}_{\text{c}}\left(\text{x},\text{y}\right)=\sum_{{\text{k}}}{\text{w}}_{{\text{k}}}{\text{f}}_{\text{k}}\left(\text{x},\text{y}\right). $$

### Outcomes and validation

Our primary outcome was a favourable performance in detecting intussusception in our data sets. In the internal validation test, we used the AUC, highest accuracy, and highest Youden index to measure performance^32^. Accuracy measures the fraction of correct predictions over the total number of predictions. The Youden index is defined as sensitivity + specificity – 1, that is, the vertical distance between the 45° line and the point on the ROC curve. In the external validation tests, we selected the optimal cut-off value based on the highest Youden index value^33^ from the internal validation tests; this was done because plain abdominal radiography is commonly used as a first-line screening test for intussusception in patients with gastrointestinal signs and symptoms. Furthermore, we applied the cut-off values in the external validation to determine the AUC and Youden index values.

### Statistical analysis

All the data were compiled using a standard spreadsheet application (Excel 2016; Microsoft, Redmond, WA, USA) and analysed using NCSS 12 (Statistical Software 2018, NCSS, LLC. Kaysville, Utah, USA, ncss.com/software/ncss). The Kolmogorov–Smirnov test was used to verify that all data sets had a normal distribution. We generated descriptive statistics and presented them as frequencies and percentages for categorical data, and as medians and interquartile ranges, (IQR) (non-normal distribution), means and standard deviation (SD) (normal distribution), or 95% confidence intervals (95% CI) for continuous data. The independent t-test or the Kruskal–Wallis test was used to compare the positive and negative groups. Categorical variables were presented as numbers and percentages and analysed using a chi-square test. Two-tailed p-values < 0.05 were considered statistically significant. We used a single ROC curve and cut-off analysis for the internal test and two ROC curves with the independent groups design for comparing the ROC curves of the external and internal validation tests. Two-tailed p-values < 0.05 were considered statistically significant.

## Abbreviations

CNN: Convolution neural network
AUC: Area under the receiver operating characteristic curve
ROC curve: Receiver operating characteristic curve
DICOM: Digital imaging and communication in medicine
PACS: Picture archiving and communication system
SSD: Single shot multibox detector
ROI: Regions of interest
SGD: Stochastic gradient descent
CAD: Computer-aided diagnosis
CAMs: Class activation map
IQR: Interquartile ranges
SD: Standard deviation
CI: Confidence interval
